# Optimism as a Prior Belief about the Probability of Future Reward

**DOI:** 10.1371/journal.pcbi.1003605

**Published:** 2014-05-22

**Authors:** Aistis Stankevicius, Quentin J. M. Huys, Aditi Kalra, Peggy Seriès

**Affiliations:** 1 Institute for Adaptive and Neural Computation, University of Edinburgh, Edinburgh, United Kingdom; 2 Translational Neuromodeling Unit, Institute for Biomedical Engineering, University of Zürich and ETH Zürich, Zürich, Switzerland; 3 Department of Psychiatry, Psychotherapy and Psychosomatics, University Hospital of Psychiatry Zurich, Zürich, Switzerland; 4 Gatsby Computational Neuroscience Unit and Wellcome Trust Neuroimaging Centre, UCL, London, United Kingdom; Hebrew University, Israel

## Abstract

Optimists hold positive *a priori* beliefs about the future. In Bayesian statistical theory, *a priori* beliefs can be overcome by experience. However, optimistic beliefs can at times appear surprisingly resistant to evidence, suggesting that optimism might also influence how new information is selected and learned. Here, we use a novel Pavlovian conditioning task, embedded in a normative framework, to directly assess how trait optimism, as classically measured using self-report questionnaires, influences choices between visual targets, by learning about their association with reward progresses. We find that trait optimism relates to an *a priori* belief about the likelihood of rewards, but not losses, in our task. Critically, this positive belief behaves like a probabilistic prior, i.e. its influence reduces with increasing experience. Contrary to findings in the literature related to unrealistic optimism and self-beliefs, it does not appear to influence the iterative learning process directly.

## Author Summary

The optimism bias is regarded as one of the most prevalent and robust cognitive biases documented in psychology and behavioral economics. In individuals, trait optimism is usually measured using self-report questionnaires. However, choices in simple behavioral tasks can also be used to infer how optimistic people are in practice. We asked human subjects to fill in questionnaires about trait optimism, then to participate in a behavioral experiment where they needed to infer the likelihood of visual targets to be associated with a reward. Using modeling, we could then quantify the link between self-report trait optimism and decision or learning biases. We find that people who report that they are optimistic have a positive *a priori* bias on the likelihood of future reward, whose influence reduces with experience. In our task, trait optimism doesn't distort how new information is integrated: subjects update their estimates similarly following information that is better or worse than expected.

## Introduction

Optimism is known to play an important role in human experience leading to more happiness, greater achievements and better health [1], although inappropriate optimism can also lead to poor choices [2]. Low optimism is also closely associated with depression and anxiety [3]. Because of this, there has been a recent surge of research interest so as to unveil the underlying mechanisms of optimism, its neural substrate and behavioral consequences [4].

Trait optimism is generally measured using questionnaires, the most common of which is the Life Orientation Test-Revised (LOT-R) [5]. The LOT-R is a series of six statements (and an additional four filler items) with four positively and four negatively worded items (e.g. “In uncertain times, I usually expect the best” and “If something can go wrong for me, it will”) which subjects have to score from 0 to 4 according to how much they agree with it.

Optimism is thought to affect cognitive processes in at least two ways. First, it biases one's expectations in a positive direction: while optimists view the glass as being half-full, pessimists might perceive it as half-empty. Formally, such a divergence in the interpretation of the same object could result from the influence of different prior beliefs. Second, optimism also appears to impact learning: optimists sometimes maintain positive beliefs in defiance of what should be strong evidence, such as doctors underestimating the risks of treatments or people continuing to buy lottery tickets. Recent work has shown that this may be due to biases towards more readily learning from “good news” (i.e. outcomes that are better than expected) than from “bad news” [6]–[9]. This biased learning could serve as a way to maintain the biases on the beliefs themselves. However, it is not known whether this impact of optimism on learning generalizes to all settings or is only restricted to the domain of personally relevant information and self-beliefs. More generally, how optimism relates to measurable cognitive biases is still poorly understood.

To approach these questions, we designed a behavioral task in which positive beliefs about future outcomes as well as learning biases could be quantified in individuals, independently from LOT-R scores and subjective introspection. This paradigm allowed us to disambiguate whether trait optimism functions as a prior belief on the likelihood of future outcomes, as a learning bias, or both.

## Results

Fifty-one subjects took part in the main study (30 males and 21 females, age range: 17–45 years old). They were first asked to answer a set of questionnaires assessing trait optimism and related personality traits: the LOT-R as a measure of trait optimism [10], the Barratt Impulsiveness scale [11], NEO five-factor inventory [12], Digit Span AB task [13] and MINI International Neuropsychiatric Interview questionnaire [14].

**Figure 1:**
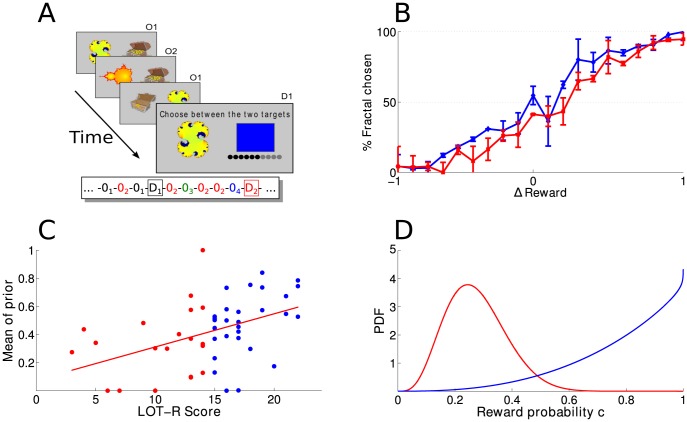
a) Cartoon of the task: subjects are presented with a sequence of stimuli (here: O_1_, O_2_, O_1_) followed by a decision screen (D_1_). Here the subject needs to choose between the yellow fractal and the square for which the reward probability is given by the number of blue dots (6 dots, indicating a probability of 60%). Inset: Example of a longer sequence of interleaved observation screens and decision screens. **b**) Performance of the subjects (% trials in which they chose the fractal stimulus) as a function of the difference between the observed reward rate of the fractal being considered and the reward probability of the square. Compared to pessimistic people (red, LOT-R≤mean LOT-R), optimistic people (blue, LOT-R>mean LOT-R) tend to overestimate the probability of reward associated with the uncertain fractal stimulus. Errors bars denote standard deviation. **c**) Correlation between subjects' LOT-R scores and the mean of their prior distribution p(c) that the fractal stimulus will lead to a reward (r = 0.438, p = 0.001). **d**) Examples of the prior distributions that were extracted for subjects 10 (pessimistic, LOT-R = 3) and 11 (optimistic, LOT-R = 22) based on their task performance.

The subjects then performed the behavioral task. Each trial started with the presentation of one of many fractal conditioned stimuli (CS). This was followed by a binary outcome (reward, depicted as a full treasure chest or no reward, empty chest) with a probability c_i_ drawn uniformly between 0 and 1, that was fixed for each fractal CS but unknown to the subjects (Figure 1a, see Methods). Each CS was presented only a few times (four times on average) and presentations were interleaved. After a fractal CS had reached its allotted number of presentations, subjects were asked to choose between the fractal CS and a colored square to maximize their chance of getting a reward. They were instructed that the reward probability of fractal CSs was constant and as experienced so far. The reward probability of the colored square was indicated explicitly by the dots underneath it. Subjects were not given feedback about their instrumental choices but told that the outcomes would determine their final score.

Because subjects were given very little information about the true CS reward probability, we expected their reward expectations for fractal CSs (but not for the colored squares) to reflect both the information they had been exposed to, and subjects' prior beliefs about the probability of rewards. By varying the number of presentations before the instrumental choice point, we could probe the learning process at various time points. We asked three questions: i) Does trait optimism relate to a prior belief about the probability of reward c_i_ associated with each fractal stimulus? ii) Does this influence fade with increasing experience, as it should if optimism works as a prior belief, or is maintained or even amplified, as it should if optimism affects learning? iii) Are the effects about prior beliefs valence specific i.e. do they correspond only to an overestimation of the likelihood of positive events or also to an underestimation of the likelihood of aversive events?

Optimistic participants (i.e. with LOT-R>mean LOT-R) were biased towards overestimating the probability that rewards would follow fractal CSs (Fig. 1b).

To ascertain whether this bias was due to a prior belief related to optimism, we modeled the task as an optimal Bayesian inference process. Subjects were assumed to optimally combine the binary evidence p(D_i_|c_i_) regarding the probability of the observations D_i_ (number of rewards observed over trials) given some probability of reward for that fractal c_i_, with their prior expectations p(c_i_) that a reward would be given (see *Methods*). The shape of the prior (Beta distribution) was controlled by 2 parameters: α and β, with the prior mean being 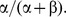 Choices were modeled as involving the comparison of the reward probability associated with the square with the estimated mean of the posterior distribution p(c_i_|D_i_), which describes the subjective belief about a reward being associated with each fractal. The variability in the decision process was parameterized by a softmax temperature parameter γ. These parameters were estimated for each subject based on their performances at the task, using Maximum Likelihood. Each participant was thus described by 3 free parameters: α, β and γ.

Table 1 shows the correlations between personality traits as measured by the different questionnaires and the parameters extracted from the behavioral task (mean of the prior: α/(α+β) and γ). Interestingly, across all participants, the average prior mean was slightly (but statistically significantly) lower than 0.5, i.e. slightly pessimistic according to this measure. However, when participants were divided into two groups, “optimists” or “pessimists” based on whether their LOT-R scores were higher or lower than the average LOT-R score for the experiment, we found that the mean of the prior differed significantly between the two groups, with optimists having a higher prior mean than pessimists. More generally, the mean of the prior correlated significantly with the LOT-R score (Figure 1c) and not with any other personality trait. This correlation remained significant after Bonferroni correction for 42 comparisons (p = 0.042). Thus, in this task, subjects' *a priori* beliefs about reward probabilities were selectively and parametrically related to their LOT-R optimism score.

**Table 1:** Correlations between personality traits as measured by the different questionnaires and parameters extracted from the behavioral task.

	α/(α+β)	LOT-R	Ne	Ex	Op	Ag	Con	Att.	Motor	S-C	C-C	P	C-In	TI	DSAB
**γ**	0.190	−0.076	0.117	−0.166	−0.094	0.022	−0.184	0.112	0.025	0.243	0.194	0.216	0.204	0.257	−0.031
**α/(α+β)**		*0.438	−0.295	0.285	0.147	0.205	0.295	−0.106	0.031	−0.152	0.061	0.061	−0.092	−0.061	−0.003
**LOT-R**	*0.438		*−0.453	**0.533	**0.308**	−0.065	0.287	−0.126	**0.375**	0.007	−0.205	−0.128	0.106	0.047	−0.110

Values in bold have correlation coefficients higher than 0.3. Values marked with ** have p-values less than 0.01, values marked with * have p-values less than 0.05. These p values are obtained after applying the Bonferroni correction for 42 comparisons. Two subjects had one/several disorders ticked in the M.I.N.I., however their results do not show any significant differences from other subjects' results. The correlation between LOT-R and neuroticism and extraversion has been reported before [15].

Ne = Neuroticism, Ex = Extroversion, Op = Openness, Ag = Agreeableness, Con = Conscientiousness, Att = Attention, Mot = Motor, S-C = Self-Control, CC = Cognitive Complexity, P = Perseverance, CI = Cognitive instability, TI = Total Impulsiveness, DSAB = Digit Span AB score, and LOT-R = Life Orientation Test - Revised.

To ascertain more directly whether optimism might also relate to the learning process, we fitted reinforcement learning models to the behavior. These models describe the learning process explicitly by assuming that subjects maintain an estimate of the value *V* of each fractal *c_i_*, and update this iteratively by adding the prediction error – the difference between the assumed value and the observed outcome [16]. The models had an initial value *V*_0_ which plays a role similar to the prior mean belief 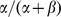 in the Bayesian model. In addition, the models allowed for selective learning biases by having two learning rates: ε_+_ for better than expected and 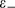 for worse than expected outcomes. These models gave a less parsimonious account of the data than the Bayesian model (worse BIC values– see *Methods*) and LOT-R scores correlated with the initial value *V*_0_ (p = 0.002, r = 0.541 in Model RL_b_), but not with either of the learning rates ε_+_ or ε_−_ or the difference between them (all p>0.1). Moreover, models that did not allow for subject-specific *V*_0_ did not capture performance differences between optimists and pessimists. Thus, optimism is well described in terms of a positive prior belief on the likelihood of reward, and does not appear to affect the learning process.

### Control experiment: Reducing the uncertainty

If optimism really functions like a prior, then its influence should fade the more subjects are given evidence about the association of stimuli and reward. If the amount of evidence is sufficiently large then subjects' performance should become independent of their prior biases. For our Bayesian analysis, this means that the simplest model that would describe their performance is one with a non-informative prior. On the contrary, if optimism affects learning, the difference between optimists and pessimists should be maintained or even amplified with experience. We conducted a control experiment aimed at testing this directly. This experiment also excluded a potential confound in the previous experiment, namely that optimistic subjects might have an *a priori* preference for fractals. The second experiment was identical to the first one, except for two changes in experimental parameters, designed to reduce the level of uncertainty in the task:

- The average number of times a given fractal was shown before a decision was requested was increased from 4 to 10;
- Instead of being interleaved, the fractals were now presented in blocks.

**Figure 2:**
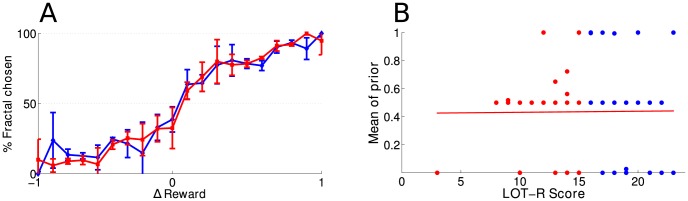
Reduced uncertainty experiment. **a**) Performance of the subjects (percentage of trials in which they chose the fractal stimulus) as a function of the difference between the observed reward rate of the fractal being considered and the reward probability of the square. Pessimistic (red, LOT-R≤mean LOT-R) and optimistic people (blue, LOT-R>mean LOT-R) behave similarly. Errors bars denote standard deviation. **b**) Correlation between subjects' LOT-R scores and the mean of their prior distribution p(c) that the fractal stimulus will lead to a reward (r = 0.009, p = 0.95).

A total of 51 new participants (28 males and 23 females, age range: 17–46 years old) participated in this version of the experiment. One subject (male) was post-hoc excluded from further analysis, because he did not achieve a 50% performance. In line with our hypothesis, we found that under those conditions, the difference of performance between optimistic and pessimistic subjects disappeared (Figure 2).

No correlation was found between the LOT-R score of individual subjects and the mean of their prior (r = 0.009, p = 0.95; a correlation significantly different from that of experiment 1: Fisher's Z = 2.26, p = 0.02; achieved power: 1−β = 0.87 assuming the effect size is the same as in experiment 1). The shape of the individual priors extracted from the subjects' performance was always close to a non-informative (i.e. Jeffrey's) prior (α = β = 0.5). In fact, in this control experiment, contrary to the main experiment, model comparison (BIC) shows that the performance of every single subject was better described by the simpler model in which the prior is chosen to be fixed and non-informative rather than by a prior with flexible α and β (vs. 45% of the subjects for experiment 1). This suggests that, in this case, subjects were able to correctly take into account the evidence and override their prior expectations: they now behave in a way indistinguishable from that of having unbiased prior beliefs.

Furthermore, the reinforcement learning models again failed to account for the data better than the Bayesian models, while supporting similar conclusions: the LOT-R score correlated neither with the learning rates 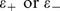 nor with the initial value *V_0_* (all p>0.1). Table 2 and 3 present the group averages of the best-fitting parameters for all the models.

**Table 2:** Best-fitting parameters for the Bayesian model summarized per experiment and averaged for the entire group of subjects and per subgroup (optimists and pessimists).

	Group	LOT-R	Bayesian Model		Sig.
			α/(α+β)	γ	
**Experiment 1**	**Mean**	14.70 (4.42)	0.42 (0.23)	7.88 (3.93)	
	**Optimists (N = 31)**	17.30 (2.36)	0.47 (0.21)	8.01 (4.09)	*/*/n.s
	**Pessimists (N = 20)**	10.60 (3.69)	0.33 (0.25)	7.75 (3.78)	*/*/n.s
**Experiment 2**	**Mean**	15.65 (4.27)	0.49 (0.37)	4.03 (1.64)	
	**Optimists (N = 26)**	18.96 (2.30)	0.50 (0.40)	3.82 (1.69)	*/n.s./n.s
	**Pessimists (N = 21)**	12.33 (4.02)	0.48 (0.38)	4.18 (1.61)	*/n.s./n.s
**Experiment 3**	**Mean**	15.44 (3.60)	0.56 (0.32)	6.71 (5.30)	
	**Optimists (N = 30)**	18.32 (3.13)	0.56 (0.37)	6.76 (4.62)	*/n.s./n.s
	**Pessimists (N = 20)**	12.55 (3.87)	0.55 (0.31)	6.67 (5.40)	*/n.s./n.s

Each column presents the mean value, with the standard deviation between brackets. Significance of the differences is shown on the right of the table: an asterisk in the corresponding column (left to right: LOT-R; α/(α+β) which defines where the prior is centered; γ is the softmax decision parameter) indicates a p value less than 0.05 for a t-test between optimists and pessimists.

**Table 3:** Best-fitting parameters for the RL models summarized per experiment and averaged per group.

	Group	LOT-R	RL Models				Sig.
			ε_+_	ε_−_	V_0_	τ	
**Experiment 1**	**Mean**	14.70	0.09/0.08/0.09/X	X/0.12/0.13/X	X/X/0.46/0.42	3.63/3.64/3.65/3.57	*/n.s/n.s/*/n.s.
	**Optimists (N = 31)**	17.30	0.09/0.11/0.08/X	X/0.14/0.14/X	X/X/0.54/0.47	3.79/3.80/3.77/3.76	*/n.s/n.s/*/n.s.
	**Pessimists (N = 20)**	10.60	0.09/0.07/0.10/X	X/0.08/0.11/X	X/X/0.35/0.33	3.34/3.38/3.49/3.46	*/n.s/n.s/*/n.s.
**Experiment 2**	**Mean**	15.65	0.27/0.25/0.22/X	X/0.30/0.27/X	X/X/0.61/0.56	2.21/2.31/2.48/2.44	*/n.s/n.s/n.s./n.s.
	**Optimists (N = 26)**	18.96	0.27/0.24/0.21/X	X/0.27/0.24/X	X/X/0.64/0.59	2.28/2.37/2.52/2.51	*/n.s/n.s/n.s./n.s.
	**Pessimists (N = 21)**	12.33	0.26/0.26/0.22/X	X/0.32/0.29/X	X/X/0.59/0.54	2.15/2.24/2.46/2.39	*/n.s/n.s/n.s./n.s.
**Experiment 3**	**Mean**	15.44	0.50/0.51/0.48/X	X/0.50/0.42/X	X/X/0.59/0.57	0.38/0.53/0.52/0.51	*/n.s/n.s/n.s./n.s.
	**Optimists (N = 30)**	18.32	0.46/0.50/0.45/X	X/0.49/0.39/X	X/X/0.68/0.65	0.48/0.61/0.63/0.59	*/n.s/n.s/n.s./n.s.
	**Pessimists (N = 20)**	12.55	0.52/0.51/0.49/X	X/0.52/0.44/X	X/X/0.53/0.52	0.33/0.48/0.47/0.41	*/n.s/n.s/n.s./n.s.

Each value reported in the column shows mean values for different RL models (left to right: RL_ε_; RL_2_; RL_2b_; RL_b_) and X means that the variable is not used in a model. Significance of the differences is shown on the right of the table: an asterisk in the corresponding column indicates a p value less than 0.05 for a t-test between optimists and pessimists. Parameters ε+, ε− denote the learning rates for positive and negative errors respectively, v_o_ is the initial value of all CS, and τ is the softmax decision parameter.

In view of these results and so as to test whether the dependency of the bias with level of uncertainty could also be observed in the same group of participants (vs. between two different groups), we also re-analyzed the data of experiment 1. We compared performances (% choices) for the fractals that were “over-observed” (observed more than 4 times) compared to the fractals that were “under-observed” (less than 4 times). We tested whether optimists and pessimists differed in their “under-observed” and “over-observed” biases using two sample t-tests. Consistent with our hypothesis, we found that the differences in performances between optimists and pessimists was statistically significant for “under-observed” fractals (p<0.01), but not for the “over-observed” ones (p = 0.135). We used a two-sample, one-tailed t-test to test if one effect is significantly greater than the other, and found that this was the case (p = 0.0017).

### Punishment avoidance experiment

We finally asked whether optimism could also predict prior beliefs about the likelihood of losses, by repeating the experiment with punishments (i.e. losses of points) rather than rewards. The experimental procedure was the same as in Experiment 1, except for the fact that, here, both the CS and the square stimuli were associated with a probability of punishment (instead of reward), depicted by a cartoon of a sad face. Subjects were now asked to estimate the probability of punishment c_i_ associated with the CS and to avoid punishment when choosing between the CS and the square stimulus.

**Figure 3:**
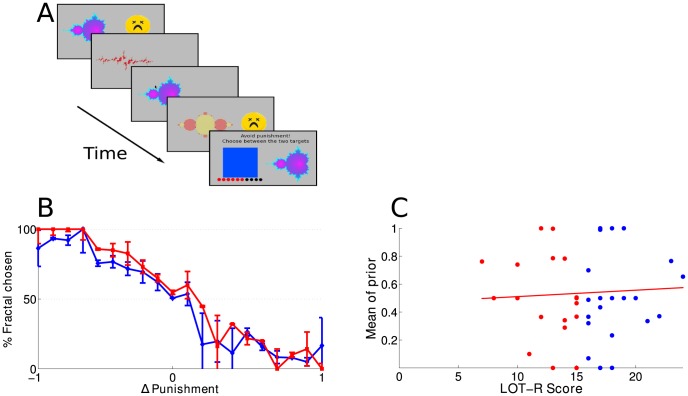
Punishment avoidance experiment. **a**) Cartoon of the task. The CS can either lead to a punishment (indicated by a sad face) or nothing. **b**) Performance of the subjects (percentage of trials in which they chose the fractal stimulus) as a function of the difference between the observed reward rate of the fractal being considered and the reward probability of the square. Pessimistic (red, LOT-R≤mean LOT-R) and optimistic people (blue, LOT-R>mean LOT-R) behave similarly. **c**) Correlation between subjects' LOT-R scores and the mean of their prior distribution p(c) that the fractal stimulus will lead to a reward (r = −0.049; p = 0.74).

A total of 51 subjects (29 males and 22 females, age range: 17–38 years old) participated in this version of the experiment. Four subjects (1 female and 3 males) were post-hoc excluded from further analysis, because they did not achieve a 50% performance. We found that under those conditions, optimistic and pessimistic subjects had similar performances (Figure 3). Moreover, subjects' prior mean did not correlate with the LOT-R score (r = 0.049, p = 0.74; significantly different from that of experiment 1, Fisher's Z = 2.05, p = 0.04 and achieved power: 1−β = 0.86). The RL models were not as good at explaining the data as the Bayesian model (in terms of their BIC values, see *Methods*) and the extracted model parameters didn't differ between groups (Table 2 and 3).

## Discussion

In conclusion, trait optimism as measured by the LOT-R questionnaire is found to correlate with performance biases in a simple Pavlovian conditioning task: optimistic subjects over-estimate the probability of reward associated with the uncertain target. This bias affects the estimation of future rewards but not of future losses in our task. It conforms to Bayesian principles of optimal inference and disappears when the level of uncertainty decreases.

Our findings are consistent with intuition about the nature of optimism in humans, as well as evidence that optimistic people are more likely than pessimists to have positive gambling expectations [17]. Interestingly in our study the observed estimation biases concern future outcomes for *neutral stimuli* (the fractal shapes). This can be contrasted with studies looking at unrealistic optimism, which concern self-beliefs. Unrealistic optimism has been defined as the “favorable difference between the risk estimate a person makes for *him or herself* and the risk estimate suggested by a relevant objective standard” [18]. Compared to such studies, our findings differ in two ways. First, unrealistic optimism studies show that participants are biased in their estimates of positive outcomes (such as graduating from college, getting married, having a favorable medical outcome) but even more so in their estimates of negative outcomes (suffering from a disease, getting divorced etc.) [18]. In our study, on the other hand, optimism corresponded to an overestimation of the probability of positive outcomes (reward), but not to an underestimation of the probability of negative outcomes (punishment, experiment 3). This was unexpected at first, since the LOT-R contains statements related to predictions of both positive and negative events. One possible reason might be that the salience of positive and negative outcomes may have differed. Future studies will be needed to assess the generality of the asymmetric effect of optimism, in particular by using more salient negative reinforcers.

Second, in our experiment, participants don't seem to be biased in the learning process itself. Optimists and pessimists differ in their initial biases but not in how they accumulate new information. Moreover, fitting the data with reinforcement models showed that they learned similarly from positive prediction errors (“good news”) and negative prediction errors (“bad news”). Studies looking at updating of beliefs related to one's personal qualities or future life events [6]–[9], on the other hand, have typically found that people are likely to discount new information that is worse than their current beliefs, and as such appeared to be “non-Bayesian” learners. For example, Eil and Rao (2011) find that participants tended to discard negative information (“bad news”) when processing personal information regarding their IQ or Beauty, whereas “good news” led to a much tighter adherence to Bayesian updating of their beliefs [7]. Wisfall and Zafar (2011) also conclude that college students in their study are not Bayesian updaters when they have to form and update their beliefs about their future earnings [9]. Similarly, in a task where participants have to estimate the likelihood of a negative future life event, such as divorce or cancer, Sharot et al (2011) show that participants updated their beliefs more in response to information that was better than expected compared to information that was worse [6].

There are many important differences between the current paradigm and those studies, which makes the comparison difficult. As stated above, a crucial difference is whether the quantity to be estimated concerns the self or a neutral stimulus. This can lead to large differences in motivation in the learning process: when information is personally relevant, participants have a motive to disregard negative information so that they can keep a rosy view of the future. In our task, on the other hand, there is no intrinsic advantage of keeping a biased estimate for the probability of rewards associated with the fractals. Consistent with this idea, Eil and Rao found that participants conformed Bayesian rationality in their control (neutral) condition [7]. Mobius et al. provide a theoretical framework that can possibly unify all these results: they suggest that the updating asymmetry itself can be explained by Bayesian principles in a model where agents derive utility from their beliefs. This model includes the fact that believing that one has a higher than average IQ, for example, even if it is untrue, has an intrinsically “rewarding” value, in that it helps self-confidence [8].

Other differences in experimental design between these studies and ours are worth mentioning. In [6], for example, the information given about the occurrence probability (e.g. “actual likelihood cancer 30%”) is explicit, high-level, provided only once and open to interpretation (i.e. participants can decide whether this should apply to them or not) whereas our study involves actual experienced outcomes that have to be integrated over time for the occurrence probability to be estimated. Despite these differences, our results combined with those mentioned above suggest that that there might be at least two distinct computational expressions of optimism: one, corresponding to very general initial biases for simple associations of stimuli and outcomes that can be overcome by learning; and a second one directly affecting the learning process in the domain of personally relevant beliefs with strong emotional content (such as one's qualities, future health or success). It will be important in the future to clarify the boundaries between these domains.

The experimental paradigm opens the door to a number of investigations. For example, our experimental paradigm offers new routes to the differentiation between optimism and pessimism, and optimism and hope, which are sometimes believed to be different constructs [19], [20]. There is a documented link between depression and (the lack of) unrealistic optimism [21], [22]. For example, Strunk et al investigated how participants estimate the likelihood of positive and negative future life events and found that depressed individuals exhibit a pessimistic bias by over-estimating the likelihood of negative future events [22]. It will be important to see how participants with depressive symptoms perform in our task. It will also be interesting to examine the impact of pharmacological manipulations particularly of dopamine or serotonin [23].

Finally, optimistic biases have also been reported in animals and it has been proposed that those biases could be used as an indicator of affective state [24]. For example, Harding et al have found that rats can display optimistic or pessimistic biases when interpreting ambiguous stimuli. Moreover, such biases correlated with the quality of their housing (unpredictable – which induces symptoms of a mild depression-like state – or predictable) [25]. In this context, it is interesting that our paradigm can also be adapted for use with animals. It would be very interesting to investigate the relation between cognitive biases observed in such situations of ambiguity with the ones we report. Adapting our paradigm for use with animals will also allows the translational investigation of the underlying neural substrate [26].

## Methods

### 1. Ethics statement

All participants gave informed written consent and the University of Edinburgh Ethics Committee approved the methods used in this study, which was conducted in accordance with the principles expressed in the Declaration of Helsinki.

### 2. Behavioral task

All experiments took place at the *Perception lab* at the University of Edinburgh. 51 naive subjects took part in each experiment and were recruited mainly among students of the University of Edinburgh. First, subjects were asked to sign a consent form and to fill in the questionnaires. Then, a short trial version of the behavioral task was presented, during which verbal and text instructions were given. Once subjects had confirmed that they were comfortable with the task, they were presented with the full version of the experiment.

Visual stimuli were generated using the Matlab programming language and displayed using Psychophysics Toolbox [27]; [28]. Participants viewed the display in a darkened room on a 20″ monitor at a viewing distance of approx. 100 cm. Stimulus sizes on the screen were 8×8 cm and 5×5 cm for fractals and chests respectively.

The experiment contained two types of screens (Figure 1a): i) a series of *observation screens* which subjects had to passively observe. On each of these screens a fractal stimulus (or conditioned stimulus, CS) was shown to be associated with a binary reward (the presentation of the fractal was followed after 700 ms. by the presentation of a full treasure chest) or not (the fractal led to an empty chest); intermixed with ii) 60 *decision screens*, where the subject was asked to choose between a fractal stimulus that he had observed before and a blue square, by clicking on it with the mouse. The task of the subjects was to maximize reward gain.

More precisely, there were 60 different fractal stimuli in total. They were generated using Matlab code available from the C.I.R.A.M. Research center in Applied Mathematics at the University of Bologna. The probability c_i_ for each fractal CS to lead to a reward was drawn randomly between 0 and 1 at the start of the experiment and kept unknown to the subject. As described above, CSs were then shown in random sequences of observation and decision screens. More precisely, in the main experiment, each CS was assigned to a group of 5 fractals and those were presented in randomly interleaved observation screens before they were shown in decision screens (Figure 1a – inset). In the main experiment, each CS was observed on average 4 times before it appeared on a decision screen (the exact number was drawn from a Poisson distribution with mean 4 and truncated to be greater than 2). Each CS was involved in only one decision screen. On each decision screen, the reward probability of the square stimulus was drawn randomly from 0 and 1 (binned with steps of 0.1). This probability was explicitly indicated to the subjects, and depicted as a proportion of full circles out of a set of 10 circles (Figure 1a). The side on which the CS appeared in the decision screen was chosen randomly on each trial. Decision screens were displayed until the subject chose one stimulus by clicking on the mouse. The behavioral experiment lasted about 30 minutes.

Feedback was not given after each decision screen but each subject was given a final score at the end of the experiment. Due to funding changes, the first 42 subjects of experiment 1 were unpaid but participated in a draw with a £20 voucher prize, while subjects of experiment 2 and 3 and the last 11 subjects in the main experiment were paid £6 for participation (unrelated to their performance at the task). No significant differences were found between paid and unpaid participants' performances.

### 3. Bayesian model

We assumed that subjects behave as Bayesian observers, and estimated the probability of reward, denoted c_i_, associated with a given fractal i by computing the posterior distribution p(c_i_ |D_i_), using Bayes rule:
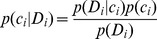
(1)where D_i_ denotes the series of observations related to fractal *i* (series of rewards observed, or not, on all observed trials) and *p(c_i_)* denotes the subject's prior belief that CS *i* will be associated with a reward.

We further assumed that subjects formed their decision by extracting the mean of this posterior distribution so as to obtain an estimate ĉ_i_ of c_i_:
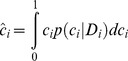
(2)We modeled the prior distribution *p(c_i_)* using a beta distribution, which is the conjugate prior of the binomial distribution. This prior has the form:
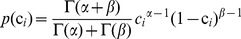
(3)where *Γ* denotes the gamma function and parameters α and β control the shape of the prior and are assumed to be the same for all CSs. A prior centered on values lower than chance was considered as a ‘pessimistic prior’, whereas a prior centered on values greater than chance was considered as an ‘optimistic prior’ in the experiment (Fig. 1d). Under this model, it can be shown that, for each fractal, the posterior mean ĉ_i_ is:
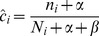
(4)where *N_i_* is the number of time fractal i was shown, and *n_i_* the number of times it was associated with a reward in the observation screens.

We assumed that subjects' decision results from a ‘softmax’ comparison between their estimate *ĉ_i_* of the probability that the CS should lead to a reward with the probability *b_i_* (explicitly given) that the square stimulus should lead to a reward on trial t. Subjects would then choose the CS with probability *p(choose fractal)*:
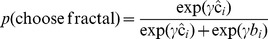
(5)where parameter γ controls how closely the subjects' responses follow the internal estimates and is assumed to be fixed during the whole session. Under this decision-making model, each subject was thus described by 3 free parameters: α, β and γ. These parameters were estimated for each subject based on their task performances, using Maximum Likelihood and numerical optimization methods in Matlab (fmincon).

### 4. Reinforcement learning models

We also fitted various reinforcement learning (RL) models to our data. Our idea was to assess whether RL models could capture the differences in performance between optimists and pessimists in experiment 1, and if so, to identify the parameters which would explain those differences. We were particularly interested in assessing whether optimists and pessimists would differ most in the parameters governing value update as a function of the sign of the prediction error or in those parameters setting the initial biases (consistent with the alternative account of optimism as a prior belief). We used a simple temporal-difference (TD) learning algorithm. In these models, subjects learn a value V(s_i_) for each CS *i*, which is initialized at v_o_ (identical for each CS) and then updated after each observation of that CS, according to:
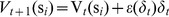
(6)where δ_t_ = r_t_−V_t_(s_i_) denotes the prediction error, r_t_ denotes the binary reward, t represents the observation number, and the learning rate ε(δ_t_) is set to hold either the same value (ε+ = ε−) for better-than-expected (i.e. δ_t_>0) and worse-than-expected outcomes (δ_t_<0), or different values (ε+≠ε−). The selection between targets 1 and 2 is governed by a softmax action selection, with additional parameter τ.
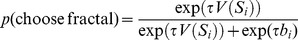
(7)where b_i_ corresponds to the reward probability of the colored square. We first examined model RL_2b_ which had 2 free learning rates ε+, ε− and free v_o_. We additionally examined simplified versions of this model, which differed in the number of parameters kept free in addition to τ:

- RL_ε_ has only one learning rate ε ( = ε+ = ε−) as free parameter, v_o_ is set to 0.5;
- RL_2_ has 2 free learning rates ε+, ε−, v_o_ is set to 0.5;
- RL_2b_ has 2 free learning rates ε+, ε−, and free v_o_;
- RL_b_ has only free v_0_, the learning rate ε ( = ε+ = ε−) is set to 0.1.

Each model was fitted to the data of each participant using maximum-likelihood estimation. Table 2 and 3 present the group averages of the best-fitting parameters for all the models.

We found that: i) only the models with free bias term v_o_ captured the difference in performance between optimists and pessimists in experiment 1 (i.e. led to significantly different parameters for optimists and pessimists); ii) in line with the hypothesis that optimism functions as a initial bias, the bias v_o_ correlated with LOT-R scores in experiment 1 (significantly so for RL_b_;: r = 0.541, p = 0.002); iii) the RL models were worse at fitting the data than the Bayesian models, both in terms of log likelihood and BIC values in all experiments (BIC for experiment 1: RL_ε_ = 71.83; RL_2_ = 74.57; RL_2b_ = 77.83; RL_b_ = 75.53, Bayesian model = 60.92; BIC for experiment 2: RL_ε_ = 80.71; RL_2_ = 83.36; RL_2b_ = 86.71;RL_b_ = 81.16, Bayesian model = 71.38; BIC for experiment 3: RL_ε_ = 90.52; RL_2_ = 93.88; RL_2b_ = 97.49;RL_b_ = 90.14, Bayesian model = 83.12). We concluded that, in our data, optimism is well described in terms of a positive prior belief on the likelihood of reward and is not significantly accompanied by selective updating during the learning process.
